# Some Notes on an Everyday Problem

**Published:** 1921-04-23

**Authors:** 


### THE HEALTH OF OUR EYES.

### Some Notes on an Everyday Problem.

In a recent lecture* on the factors which maintain the health of our eyes, Lieut.-Colonel R. H. Elliott, M.D., F.R.C.S., drew attention to a variety of simple but often unrecognised facts concerning our daily life. Myopia, or shortsightedness, is so called because people possessing this defect can see with ease objects near to the face, but are at a great disadvantage when they look at things further away from them. But many people are shortsighted for a long time before they become aware of their defect, and then they learn it, only too often, from their friends. They have no standard by which to measure their sight until their friends supply one for them. A shortsighted person gets large images, and often sees tiny objects with comparatively little effort. The man who carves the Lord's Prayer on a sixpence is always a myope; it is a much easier task for him than it would be for other people, because the sixpence appears to him about the size of half a crown.

### The Sight of Children.

But no child is *born* shortsighted. All myopia is acquired, and it is very important that we should watch over the sight of our children and limit the defect as strictly as possible. The tendency to short sight is greatest during the years of growth, and is most evident when growth is most rapid. All young people, whether myopic or otherwise, should read only good print, on good paper, and by a good light. A large number of cases of short sight commence after some illness. Influenza or measles are probably most dangerous, but any of the common infectious diseases of childhood may bring on short sight, or cause an increase of it in a patient who already suffers from this defect. What a boy or girl needs, for a time, when suffering from short sight, is a very healthy life, with nourishing food, tonics, fresh air, and the avoidance of study. Studious children should be made to take a due amount of exercise, and their reading should be so supervised that it is always carried on under favourable conditions as to light. The tendency to short sight, and especially the severer grades of it, is undoubtedly inherited, and so, whenever there is a family history of this defect, the importance of watching over the eyes of children is greatly increased.

* Delivered at the Institute of Hygiene, Wednesday, April 13.

### Some General Precautions.

The masters and mistresses of the big schools in England are much more alive to their responsibilities in this matter of caring for the sight of children than were their predecessors of a generation ago; but even to-day the lighting of schoolrooms is often very defective, and that of the studies, or "swat rooms," where preparation work is done at night, is sometimes a disgrace. Colonel Elliott reiterated that few things are more harmful to the eyes than reading bad print, and yet, he said, a good deal of the literature specially supplied for children comes under this category. Parents should choose their children's novels for them with an eye to the paper and the printing. The books of such writers as Stanley Weyman, or Anthony Hope, and of many others, can be obtained up to the required standard for visual purposes, and they will thrill the boy or girl as much as, if not more than, many of the cheap publications.

### Reading in Bed.

Is it bad to read in bed? The answer is simple. If it is done sitting up, looking down at the print, and with the aid of a good light, there is no reason why reading in bed should be any more harmful than reading at a desk. It is the position that does the mischief. The explanation of this, the lecturer said, goes back to the time, long ages ago, when the ancestor of man and the higher apes first developed the use of the opposing thumb. On that development hung the whole future of civilisation. The possession of a thumb which could be used in opposition to the fingers enabled its possessor to pick up, and so to study, the objects that lay around. The objects studied were always held below the level of the eyes, and thus, all through the ages, the muscles that depress the eyes have been more called upon than those which raise them, and to-day we find the former stronger than the latter.

If we go to the Royal Academy and look up at the pictures for two or three hours we come away with a headache. Many find the same effect after sitting in the stalls of a theatre or of a cinema house. The reason is that the eyes have been used in an unnatural position. It would be much better if the picture galleries could be arranged so that we could look down at the pictures from above. Many of those who suffer from severe headaches after visiting a picture palace can sit out a performance in comfort if they take seats in the gallery and look down on the entertainment.

